# Study on Flame Retardancy of Cotton Fabric Modified by Sulfonic Groups Chelated with Ba^2+^

**DOI:** 10.3390/molecules29225306

**Published:** 2024-11-10

**Authors:** Lingling Guo, Hongqin Lin, Zhenming Qi, Jiang Pan, Haiyan Mao, Chunmei Huang, Guoqiang Li, Chunxia Wang

**Affiliations:** 1 College of Textile and Clothing, Yancheng Institute of Technology, Yancheng 224051, China; xiaoqiaolinglong08@163.com (L.G.); lhq@ycit.cn (H.L.); qzm95916@163.com (Z.Q.); pj1090364110@gmail.com (J.P.); maohaiyan@ycit.edu.cn (H.M.);; 2 Jiangsu Yueda Home Textile Co., Ltd., 699 Century Avenue Road, Yancheng 224005, China; 15366557088@163.com

**Keywords:** cotton textile, fire resistance, sulfonic acid group, chemical bonding, chelation effect

## Abstract

A simple and innovative method was introduced for the production of green and recoverable flame-retardant cotton fabrics, where sulfonated cotton fabric (COT-SC) was synthesized by oxidizing cotton fabric with sodium periodate, followed by a sulfonation step with sodium bisulfite to provide active sites, which further chelated barium ions (Ba^2+^) to achieve flame retardancy. The morphological and structural characterizations of the fabricated cotton fabrics (COT-SC-Ba) demonstrated that the cleavage of C_2_-C_3_ free hydroxy groups within the cellulose macromolecule was chemically modified for grafting a considerable number of sulfonic acid groups, and Ba^2+^ ions were effectively immobilized on the macromolecule of the cotton fabric through a chelation effect. Results from cone calorimeter tests (CCTs) revealed that COT-SC-Ba became nonflammable, displayed a delayed ignition time, and decreased the values of the heat release rate (HRR), total smoke release (TSR), effective heat of combustion (EHC), and CO/CO_2_ ratio. TG/DTG analysis demonstrated that COT-SC-Ba possessed greater thermal stability, fewer flammable volatiles, and more of a char layer during burning than that of the original cotton fabric. Its residual mass was increased from 0.02% to 26.9% in air and from 8.05% to 26.76% in N_2_, respectively. The COT-SC-Ba not only possessed a limiting oxygen index (LOI) of up to 34.4% but could also undergo vertical burning tests evidenced by results such as the non-afterflame, non-afterglow, and a mere 75 mm char length. Those results demonstrated that the combination of SO_3_^−^ and Ba^2+^ promoted the formation of a char layer. Moreover, cotton fabric regained its superior flame retardancy after being washed and re-chelated with Ba^2+^. Additional characteristics of the cotton fabric, such as the rupture strength, white degree, and hygroscopicity, were maintained at an acceptable level. In conclusion, this research can offer a fresh perspective on the design and development of straightforward, efficient, eco-friendly, and recoverable fire-retardant fabrics.

## 1. Introduction

Fire is a double-edged sword, both a symbol of civilization and a potential threat. The risk of fire has always been considered too important an issue to ignore due to the extensive use of flammable polymer products such as textiles, plastics, and wood. Textiles are omnipresent in our lives, like bedding, sofa fabrics, carpets, and curtains, yet their inherent flammability poses significant daily fire risks [1,2,3]. Consequently, considerable efforts have been directed towards minimizing these risks and ensuring compliance with stringent fire safety regulations [4,5]. Traditionally and currently, the primary strategies for enhancing the flame retardancy of textiles have been the utilization of halogen-based, phosphorus-based, and mineral-derived chemicals (metal hydroxides, bentonite, etc.) [6,7]. However, it is now widely recognized that halogen-based flame retardants are gradually being avoided and phased out because of the hazards associated with the potential release of toxic substances. Although phosphorus-based flame retardants are widely used, their excessive use could produce more or less adverse effects on the environment and human health [8]. Therefore, developing environmentally friendly and sustainable flame-retardant methods is crucial, and significant efforts have been directed towards addressing this issue.

In this context, natural compounds deriving from various animals and plants have recently been developed. These include proteins, alginates [9], deoxyribonucleic acid [10], biomass tannins [11], chitosans, phytic acid [12], cardanol [13], etc., which are sought for their non-toxic, biocompatible, and biodegradable properties. However, there is a question to be posed regarding their cost-effectiveness before putting them into commercial production.

Inorganic materials, such as metal hydroxides and metal oxides [14], serve as flame retardants and possess numerous favorable aspects, including excellent stability, smoke suppression, and catalytic carbonization during burning [15]. Gradually, people found the majority of metal oxides and hydroxides, despite their wide use, show poor compatibility with polymers, resulting in the significant degradation of their mechanical properties when directly blended [16]. As metal salts [17] have the ability to achieve flame retardancy through chemical bonding and can also enhance the thermal stability of fabric materials by modifying them, people began to direct their focus towards them.

Li and colleagues [18] investigated the preparation and thermal degradation of sulfonated polyoxadiazole fibers that had been modified through the incorporation of metal ions, specifically Na^+^, Ba^2+^, Ca^2+^, and Mg^2+^. Among these, Mg^2+^ was capable of releasing more concentrated SO_2_ at a relatively low temperature and was able to form MgO and MgSO_4_ attached to the surfaces of the materials in comparison with other metal ions. Wu et al. [19] reported that silk fabric treated with iron, aluminum, and titanium salts at a certain concentration exhibited a self-extinguishing effect owing to the silk macromolecule’s ability to complex with metal ions. Geng and his colleagues [20] explored a method to enhance the flame resistance of poplar wood by using phytic acid–iron and epichlorohydrin–chitosan to form double-chelated metal network macromolecules (DCMCs) within the wood structure. The obtained sample showed that the DCMC was grafted onto wood through C-N bonds with an LOI of 31.9%, and the heat release rate (HRR) of DCMC–poplar could be decreased by as much as 43.4%. Inspired by alginate fiber, our team [21] modified cotton fabric with aldehyde groups and glutamic acid. This modification introduced an abundance of carboxyl groups in the form of C=N bonds, enabling the fabric to subsequently chelate calcium ions and thus obtain flame-retardant properties. Upon ignition, the embedded calcium ions facilitated the swift formation of a protective carbonaceous layer, effectively blocking the passage of flammable gasses and heat, thereby playing a pivotal role in safeguarding against fire. Therefore, metal salts, which are composed of metal elements, work as flame-retardant materials by lowering the fabric’s degradation temperature and enhancing the material’s burning performance through a catalytic effect.

Cotton, a popular natural resource, has emerged as the most adaptable material for a wide range of applications, including clothing and home furnishings as well as numerous industrial and military products, owing to its softness, comfort, exceptional water absorption, skin-friendliness, and high breathability [22,23,24]. Nevertheless, a prominent drawback of cotton lies in its heightened flammability. Therefore, it is a big challenge to improve the fire resistance of cotton and lower fire hazards in daily life.

The applications of cotton and its derivative materials, such as carboxymethyl cellulose, sulfonated cellulose, and phosphorylated cellulose [25], have led to the discovery of innovative methods in terms of removing heavy metal ions, leveraging both electrostatic attraction and chelating action. 

Elif Parlak and Özgür Arar [26] investigated the behavior and mechanisms of sulfonated cellulose in removing Cu^2+^ ions from water solutions. Their study focused on key parameters such as the adsorbing capacity, the starting pH of solutions, temperature, and the starting concentration of Cu^2+^ ions.

Dong et al. [27,28] reported sulfonated cellulose had a highly efficient adsorption to metal ions such as Fe^3+^, Pb^2+^, and Cu^2+^ in solution and could attain adsorption equilibrium in just 2 min. This was because negatively charged SO_3_^2−^ groups exhibited a tendency to interact with and bind to positive metal ions.

Choi [29] studied sulfonated cornstalk, derived from agricultural waste, for the adsorption of Ca, K, Mg, and Na from seawater. It was reported that the surface of the cornstalk acquired anionic charges after sulfonation, facilitating the adsorption of cationic ions present in seawater. In addition, the adsorption capacity for divalent cations like Ca and Mg exceeded that of monovalent cations, namely Na and K.

Wang et al. [30] converted lignin into sodium lignosulfonate through sulfonation modification to confer it excellent adsorbability and thermal stability. Previous research [28,30,31,32] has reported that sulfonated cellulose exhibits excellent adsorption properties, is easy to prepare, and has the advantage of a low cost.

Inspired by the above reports, the aim of this research is to alter the surface of cotton and improve its capacity to absorb metal ions, such as barium.

To achieve this aim, alkali-treated cotton fabrics underwent modification with sodium periodate (NaIO_4_) to produce dialdehyde carboxymethyl fabric (COT-DAC). Subsequently, the aldehyde groups were bonded with NaHSO_3_ to produce a fabric (COT-SC) that was enriched with sulfonic acid groups, thereby enhancing its Ba^2+^ adsorption capacity. This strategic modification resulted in the formation of more sturdy metal oxides/carbides/sulfides, serving as an effective non-combustible char residue barrier when subjected to heat.

## 2. Results and Discussion

### 2.1. FTIR Spectroscopic Analysis of Each Step Sample

**Figure 1:**
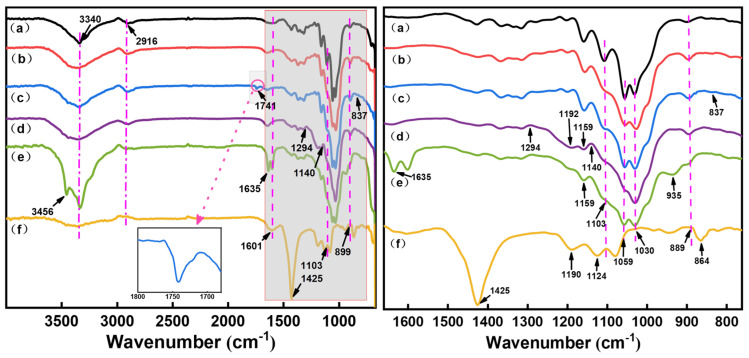
FTIR spectra (**on the left**) and their partial magnifications (**on the right**): (**a**) COT, (**b**) COT-M, (**c**) COT-DAC, (**d**) COT-SC, (**e**) COT-SC-Ba, and (**f**) the residual carbon of COT-SC-Ba after burning.

The FT-IR spectra depicted in Figure 1 showcase the various modification processes and residual carbon from vertical combustion. Compared with the original cotton sample, the vibrational peak at 3340 cm^−1^, associated with the stretching vibration from -OH [22], slowly became broader and stronger from COT-M to COT-SC-Ba. This indicates a gradual increase in the amount of hydroxyl groups on the surface of the cotton fabrics, achieved through a series of treatments, including exposure to alkaline solution, oxidation, sulfonation, and chelation reactions. Meanwhile, the characteristic peak at 2916 cm^−1^ corresponding to the symmetrical stretching vibrations of C-H bonds [26] in cellulose became slightly stronger and then weakened somewhat during the whole treatment process. Furthermore, the intensity of the peaks at 1103 cm^−1^ and 1059 cm^−1^ in pristine cotton, attributed to C-O and C-O-C bonds, respectively [33], became much weaker through alkali treatment. 

Meanwhile, a slight structure change was detected at approximately 1741 cm^−1^ and 837 cm^−1^ in COT-DAC. The carbonyl group was identified by the weaker peak at 1741 cm^−1^, whereas the peak at 837 cm^−1^ was assigned to the hemiacetal group and its hydrated forms [34,35]. Additionally, the peak near 1059 cm^−1^ belonging to C-O-C stretching vibrations [36] almost disappeared in COT-DAC, indicating that the carbon background was partially destroyed. Compared to COT-DAC, the peaks in the COT-SC sample, ranging from 1140 cm^−1^ to 1294 cm^−1^, exhibited significant changes. Specifically, the bands at 1192 cm^−1^ and 1059 cm^−1^ were assigned to the asymmetric and symmetric stretching vibrations of the S=O bonds in the sulfonic acid groups, respectively [37,38]. This suggests that the cotton fabric underwent successful sulfonation, resulting in the introduction of sulfonic groups. In addition, the characteristic peaks in Figure 1e at 1159, 1059, and 935cm^−1^ were related to the sulfonate salt groups [26,39] in the fireproof specimen. This indicates that the metal ions were successfully complexed by the effective adsorption of -SO_3_^−^.

After combustion, the distinct vibration peaks of the char layer of COT-SC-Ba were no longer present at 3340 cm^−1^, 3456 cm^−1^, 2916 cm^−1^, 1635 cm^−1^, and 1030 cm^−1^ [24], indicating the breaking of O-H, C-H, and C-O-C bonds during decomposition. Meanwhile, new absorption peaks emerged at 1425 cm^−1^, 1190 cm^−1^,1124 cm^−1^, and 864 cm^−1^ corresponding to -CO_3_^−^ [40,41], S=O, S-O, C=O, and C-S [17,42,43], which were generated by the combustion of COT-SC-Ba fabric, as illustrated in Figure 1f. It may be inferred that char residues may contain BaCO_3_, BaSO_3_, or BaSO_4_.

### 2.2. XPS Analysis

**Figure 2:**
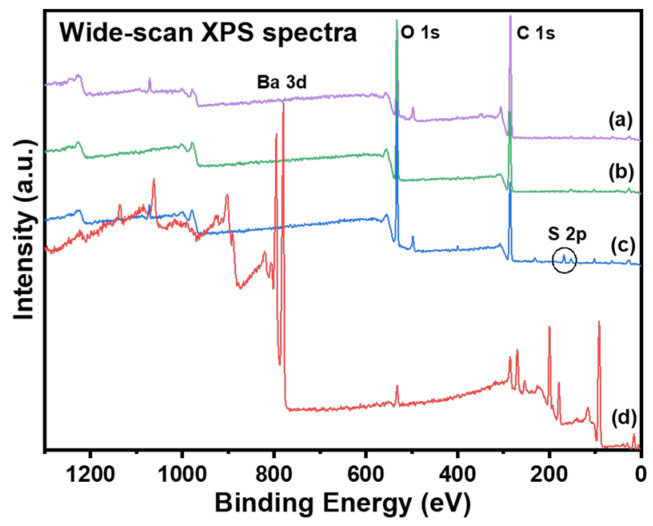
Wide-scan XPS spectra of the cotton fabric and its various treatment stages: (**a**) COT-M, (**b**) COT-DAC, (**c**) COT-SC, and (**d**) COT-SC-Ba.

The XPS spectra of the final fire-resistant cotton and each modified process sample are shown in Figure 2. From the wide-scan XPS spectra, it can be seen that all four samples displayed the presence of C and O. A new peak of S 2p was first observed in the curve of COT-SC in Figure 2c and a new peak of Ba 3d was detected in the mapping spectra of COT-SC-Ba in Figure 2d. Four elements, C, O, S, and Ba, were clearly observed in the obtained fireproof cotton fabric, indicating that the sulfur-containing groups and metal barium were uniformly distributed on the sample surface.

**Figure 3:**
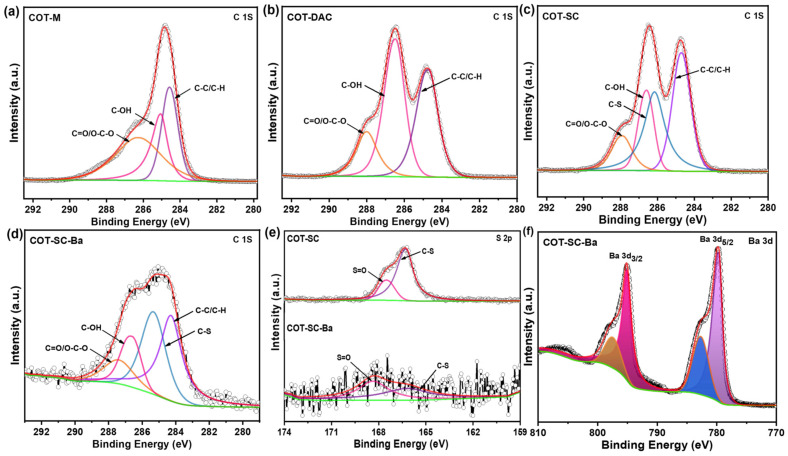
Deconvoluted high-resolution C 1s, S 2p and Ba 3d XPS spectra, from (**a**–**d**) for C 1s, (**e**) for S 2p, and (**f**) for Ba 3d, respectively.

Figure 3a–d obviously displays that there are differences between the four images regarding the characteristic C 1s peaks between 280 eV and 293 eV. For the sample treated with sodium hydroxide, it was clearly observed that there were three different peaks at energy levels of 284.6 eV, 285.1 eV, and 286.6 eV, which corresponded, respectively, to the C-C/C-H, C-OH, and C=O/O-C-O bonds [44,45,46]. Moreover, the configurations of the peaks in the C 1s spectrum of COT-DAC (Figure 3b) are remarkably distinct in contrast to the previous step specimen (Figure 3a, COT-M). The band energy for C-C/C-H centered at 284.5 eV was only significantly decreased, while the peak intensity for C=O/C-O-C not only became much smaller and weaker but also shifted obviously towards a higher binding energy, concretely, from 286.5 eV to 288.0 eV. This suggests that the C_2_-C_3_ bond underwent cleavage, and the resultant hydroxy groups were oxidized by NaIO_4_ to form aldehyde groups [47].

For the sulfonated sample, a new peak at 286.2 eV appeared in the spectrum, which was attributed to C-S bonds. Additionally, the intensity and size of the C=O peak underwent a significant reduction. These phenomena suggested that the sulfonation reaction had proceeded between aldehyde groups and NaHSO_3_, resulting in a larger amount of -SO_3_^−^ grafted onto the cotton fabric. As can also be seen from the deconvolution analysis of S 2p in Figure 3e, two distinct peaks were detected at 167.6 eV and 166.3 eV, as reported in references [42,48]: one was attributed to the inherent S=O bond in NaHSO_3_ [49], and the other was related to the sulfonation reaction where the carbons of cellulose macromolecule reacted with sulfonic acid groups to generate C-S. Thereby, it can be said that the desired design was achieved. Comparing the upper (COT-SC) and lower (COT-SC-Ba) spectra in Figure 3e, it is easy to observe that the peak for C-S was shifted towards a much lower binding energy with a 0.3 eV moving value, while the peak for S=O was shifted towards a much higher binding energy with a 0.9 eV moving value. Additionally, their intensity decreased significantly. This can be attributed to the excellent adsorption effect of the -SO_3_^−^ groups, which led to the surface of the COT-SC-Ba fabric being enveloped by a layer of barium ions. Consequently, peak vibrations became more frequent, making it difficult to detect the inner structure and elements after -SO_3_^−^ chelated with Ba^2+^. This successful chelation result could also be verified by the high-resolution Ba 3d spectrum as plotted in Figure 3f, which had the doublet peaks Ba 3d 3/2 and Ba 3d 5/2 centered at 795.2 eV and 780.0 eV, respectively, and it is the same as in previous studies [50,51]. This further shows that the final sample surface was noticeably enriched with barium, and a barium sulfite (-SO_3_Ba^1/2^) network was constructed on both the outside and inside of cotton fabric. Overall, the XPS analysis results were consistent with FTIR, and sulfonic acid groups possess an excellent adsorption ability for metal ions.

### 2.3. XRD Analysis

**Figure 4:**
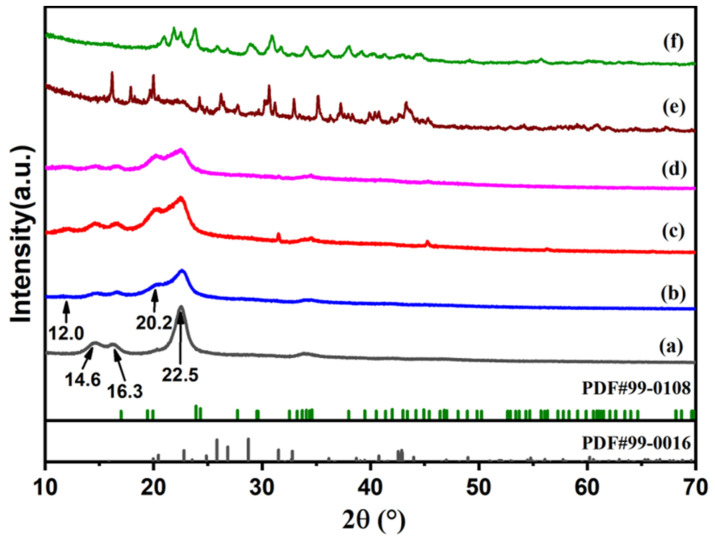
X-ray Diffraction profiles: (**a**) COT, (**b**) COT-M, (**c**) COT-DAC, (**d**) COT-SC, and (**e**) COT-SC-Ba, along with (**f**) the residual char of COT-SC-Ba.

To determine the crystallinity of samples treated with different kinds of chemical reagent and the carbon residue of COT-SC-Ba after burning, the XRD technique was employed as displayed in Figure 4. The diffraction peaks observed for pure cotton cellulose (curve (a)), corresponding to cellulose I, were consistent with previous research [52,53]. From the XRD pattern of the alkali-treated sample, it was noted that the peak positioned at 22.5°, associated with the (020) plane of cellulose II, was situated close to that of the (200) lattice plane of cellulose I [54]. Furthermore, the remarkable peak at 2θ = 20.2° assigned to the (110) plane of cellulose II was becoming progressively more prominent because of the varying degrees of preferred orientation [55]. This can be explained by a portion of the cellulose I undergoing a transformation into cellulose II through the 25 wt.% NaOH solution treatment. In comparison to curve (b), the primary peak at 2θ = 22.5° in curves (c) to (e) underwent slight variations, especially curve (e), which may be because the aldehyde groups, sulfonic acid groups, and barium ions were only introduced into the crystal surface of cellulose II [31,56] but did not change the crystalline structure [32,57]. From the shape of curve (e), it could be deduced that the interaction of Ba^2+^ ions and -SO_3_^−^ not only took place on the surface of COT-SC but also invaded the inner structure to a certain extent, which resulted in interference with the original structure [19]. In addition, curve (f) of residual carbon could further prove that cotton was completely changed through acute combustion. This result could further demonstrate that the residual carbon composition contained BaSO_4_ and BaCO_3_ by comparison with PDF#99-0108 and PDF#99-0016, which is nearly consistent with the results predicted by FTIR except for BaSO_3_. It could be explained by BaSO_3_ turning into BaSO_4_ under high temperatures. In short, these showed that cotton fabric had been successfully treated by introducing -SO_3_^−^ and Ba^2+^.

### 2.4. SEM and EDS Analysis

**Figure 5:**
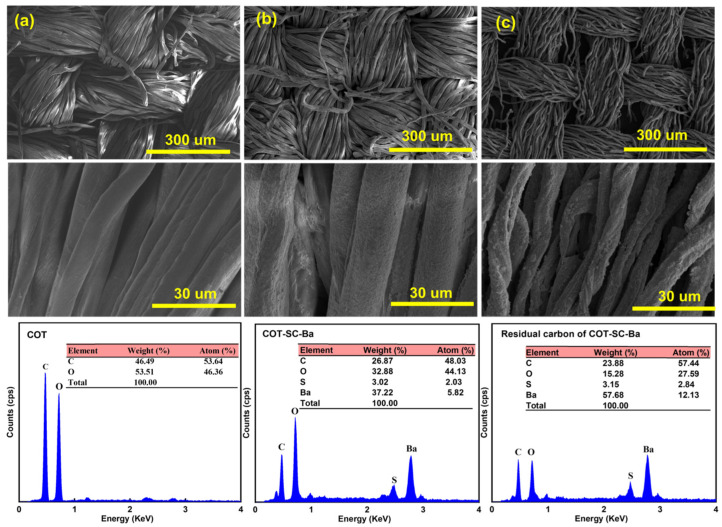
SEM images and EDS analysis before and after the fireproof modification of cotton fabric and residual carbon: (**a**) COT, (**b**) COT-SC-Ba, and (**c**) the char layer of COT-Glu-Ba fabric, which are presented with 500× and 5000× magnification in the SEM images, respectively.

Figure 5 showcases SEM images along with their corresponding EDS analysis results for several samples, including raw cotton, flame-retardant cotton, and the residual char after a vertical burning test. From Figure 5a, the pure cotton specimen exhibited a clear and, was smoother, and no other substances were found. The EDS results revealed that the raw cotton consisted of atomic percentages of 53.64% for carbon (C) and 46.36% for oxygen (O), respectively. After the raw cotton sample was sulfonated by NaHSO_3_ and then chelated with BaCl_2_, the S and Ba elements appeared on the treated cotton fabric surface, with atomic concentrations of 2.03% and 5.82%, respectively. With the appearance of S and Ba elements, a small amount of fine particle substances with high barium levels adhered to the fiber surface, rendering it no longer smooth. This indicates that Ba species were successfully chelated onto the cotton surface with the help of -SO_3_^−^. Combined with FTIR and XPS spectrum analysis, it was verified that the cotton fabric had undergone effective modification and had successfully chelated metal ions.

Figure 5c shows the burning results of COT-Glu-Ba. Despite burning fiercely, a relatively intact and uninterrupted carbon layer was maintained. The surface of the remaining carbon fiber displayed a great number of irregular bubbles, which were likely caused by the formation of vesicles as gasses released during combustion attempted to break out of the barrier formed by barium reacting with some flammable gas. The EDS analysis results also showed that the oxygen element in the fireproof cotton was consumed to a certain extent, decreasing from 44.31 wt.% to 27.59 wt.% after combustion. Meanwhile, the concentrations of Ba and S atoms increased markedly from 5.82 wt.% to 12.13 wt.% and from 2.03 wt.% to 2.84 wt.%, respectively. The elevated atomic percentages of Ba, S, and C within the carbon layer were also associated with the enhancement of effective condensed-phase flame retardancy. This effect may be induced by the thermal decomposition substance -SO_3_Ba^1/2^, as well as the subsequent formation of new substances such as BaO, BaCO_3_, and BaSO_4_ on the surface of the burning fabric. These substances were loaded on the burning sample’s surface, forming a protective film due to the reaction of Ba^2+^ with released O_2_, CO_2_, SO_2_, etc., during combustion.

### 2.5. Cone Calorimetry Analysis

**Figure 6:**
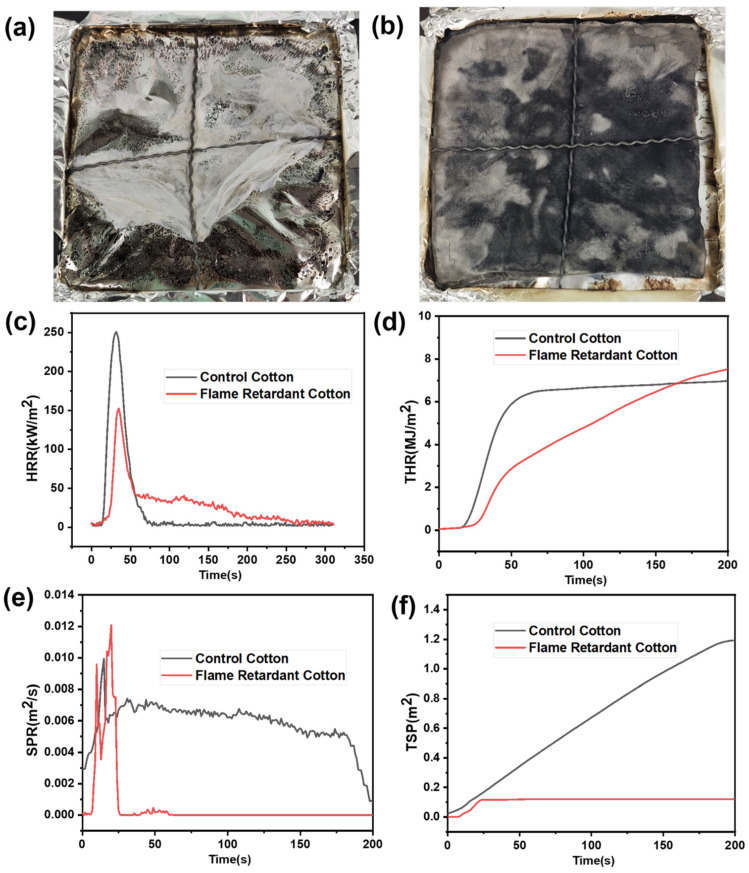
Results of the cone calorimeter test including photographs of (**a**) COT and (**b**) COT-SC-Ba, along with graphs showing (**c**) the heat release rate (HRR), (**d**) total heat release (THR), (**e**) smoke production rate (SPR), and (**f**) total smoke release (TSR).

The images and curves from the cone calorimeter test for COT and COT-SC-Ba fabric are presented in Figure 6. From Figure 6a, it can be seen that the COT fabric was completely consumed by the fire, leaving a little ash with an off-white hue. However, as displayed in Figure 6b, COT-SC-Ba fabric also underwent complete combustion, yet a fully formed char layer was left because of the Ba^2+^ ions attached to the cotton fabric surface. This was in agreement with the findings of SEM and EDS. Furthermore, according to the cone calorimeter test data listed in Table 1, the COT-SC-Ba fabric exhibited a TTI value of 23 s, which is an 8-s increase in comparison to that of COT fabric. After ignition, the COT-SC-Ba fabric reached a peak heat release rate (HRR) of 152.37 kW/m^2^ after 35 s, as depicted in Figure 6c. This maximum value was notably 98.13 kW/m^2^ less than that of the COT fabric, suggesting that it was less flammable in a fire and could significantly reduce the heat released by the fabrics during burning in comparison with the original cotton. As presented in Figure 6e, the total heat release (THR) of COT reached its maximum at about 60 s, indicating that it gathered rapidly heat in a short time. Nevertheless, the THR of the COT-SC-Ba fabric gradually rose over time and remained significantly lower than that of COT fabric, even when exposed to a high-temperature environment for approximately 180 s. This indicated that the fireproof cotton fabric exhibited greater resistance to burning. It can be traced back to the presence of metal ions, which facilitated the rapid shaping of a solid and compact char layer and also effectively hindered thermal diffusion through a barrier effect, thereby contributing to superior fire resistance. Furthermore, the peak value of the total smoke release (TSR) for COT-SC-Ba (37.02 m^2^/m^2^) significantly exceeded that of the COT fabric (1.32 m^2^/m^2^), as presented in Table 1. The reason for this was the release of more non-combustible gasses of CO_2_ and SO_2_ due to the existence of BaSO_3_ in the combustion process of the COT-SC-Ba sample, which considerably lowered the concentration of combustible gasses, hindering further burning.

As plotted in Table 1, the ratio of CO_2_ and CO (CO_2_/CO) before and after finishing was 15.36% and 11.08%, respectively. This also proved that raw cotton fabric, after being treated with a series of methods, did not ignite sufficiently during the burning process owing to the formation of a charcoal barrier [58]. The mean effective heat of combustion (EHC) of COT-SC-Ba fabric was tested to be 15.47 MJ/Kg, which decreased by 20% compared to raw cotton, indicating grafting hydrogen sulfite and chelating Ba^2+^ could have a positive effect on combustion.

### 2.6. Thermal Stability Analysis

**Figure 7:**
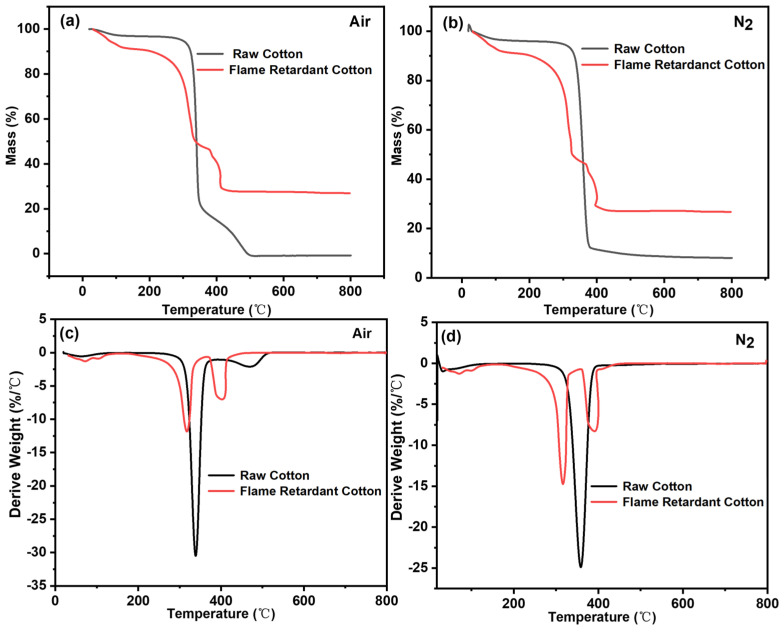
TG and DTG curves for COT and COT-Glu-Ca in (**a**) air and (**b**) N_2_ (TG), and (**c**) air and (**d**) N_2_ (DTG).

The behaviors of thermal degradation for unmodified and modified cotton fabrics have been researched through TG and DTG analysis in air and nitrogen (N_2_) environments, respectively. Relevant results are shown in Table 2. From Figure 7a,b, the final modified sample (COT-SC-Ba) began to degrade at a lower temperature with 8.6% weight loss owing to the evaporation of water absorbed and catalytic degradation by barium ions at lower temperatures (below 200 °C) [59]. However, the raw cotton fabric had almost no mass loss at the lower temperature ranging from 43 to 180 °C due to cellulose dehydration. As the temperature continued to increase, there was a rapid pyrolysis, resulting in a peak weight loss rate (Rmax) of 11.8% at 315.8 °C for COT-SC-Ba. This Rmax value is significantly lower when compared to the 18.3% Rmax observed for COT at 339.2 °C. It could be attributed to the following reasons: The grafted group -SO_3_Ba^1/2^ was decomposed slowly in the beginning stage, and Ba^2+^ ions showed a catalytic degradation effect, helping the formation of a carbon layer at a low temperature, which hindered the burning process of the cotton fabric [19]. Moreover, the main stage not only released a number of nonflammable gasses such as H_2_O, CO_2_, and SO_2_, but also resulted in the formation of an intermediate substance because of the decomposition of -SO₃^−^ groups [60], the dehydration, and the glycosidic bond breaking of modified cotton fabric [61,62]. Herein, the interaction of several reasons further slowed down the degradation of COT-SC-Ba. The residue of modified cotton fabric at 800 °C was 26.9 wt.%, whereas the residue for COT was virtually non-existent. All these confirmed that the grafted -SO_3_Ba^1/2^ groups had a substantial effect on the degradation of cotton fabric.

In N_2_, the thermal decomposition procedure of COT-SC-Ba fabric also showed three major weight loss stages. Its TG curve was basically identical to that of the original cotton fabric, while its DTG exhibited a remarkable difference between the maximum mass loss in O_2_ and that in N_2_. The third stage of decomposition occurred mainly in the range of 325.1–395.4 °C, with a mass loss of 21.06%. In contrast, when COT-SC-Ba fabric was degraded in air, it degraded in the range of 339.8–413.55 °C with a mass loss of 20.95%. The COT-SC-Ba sample exhibited a carbon residue of 26.76 wt.%. This suggests that the intermediate products further underwent a more complex decomposition, whether in an O_2_ or N_2_ environment, forming corresponding barium oxide/barium sulfate/barium carbonate, which attached to the fiber surface as a hard protective screen. The residual carbon mass in air is significantly higher than that reported in our previous work [21], indicating that the S element plays a blocking role in the pyrolysis process.

Obviously, COT-SC-Ba exhibited a lower thermal decomposition temperature, namely below 200 °C, a smaller maximum weight loss rate, and a larger amount of char residue. This can be explained by the incorporation of barium ions into the cellulosic molecules, which acts as a catalyst for the formation of additional char residue to hinder the burning of fibers in the condensed phase. In addition, sulfur elements from sulfonic acid groups have been proven to be effective flame retardants for materials in previous studies [63,64].

### 2.7. Flame Resistance Analysis

**Figure 8:**
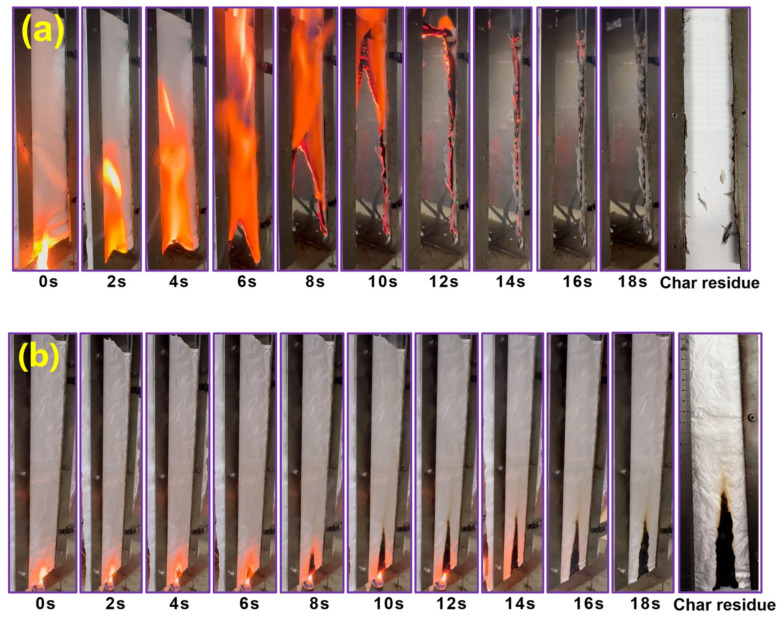

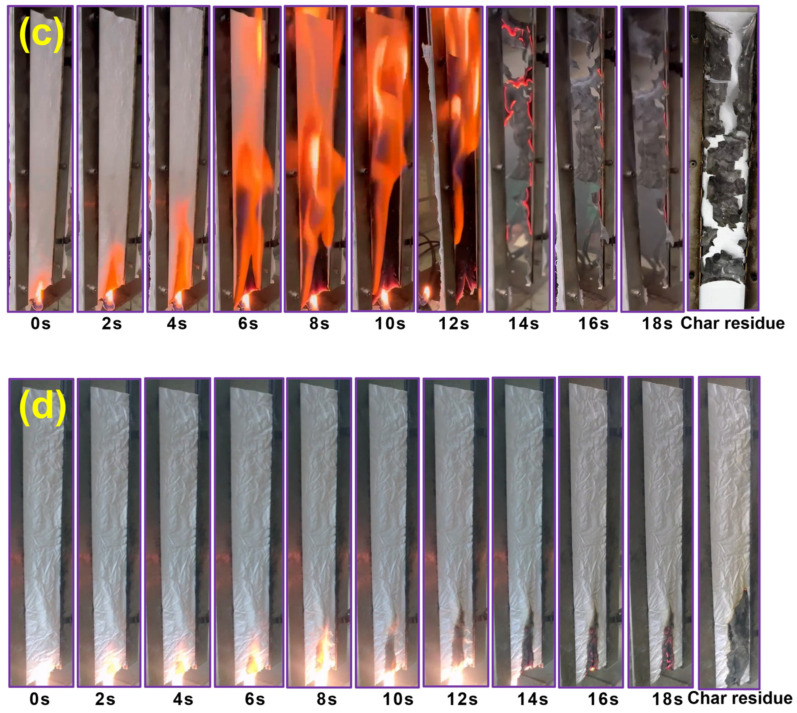
The vertical burning tests conducted on four different types of fabric at various time points: (**a**) raw cotton, (**b**) flame-retardant cotton fabric, (**c**) COT-SC-Ba after 5 washing cycles, and (**d**) washed and re-chelated COT-SC-Ba.

As displayed in Figure 8 and Table 3, the vertical burning procedure and average LOI results from three tests of various cotton fabrics, including COT, COT-SC-Ba, COT-SC-Ba washed five times, and re-chelated COT-SC-Ba, are presented. It can be visually observed that the burning properties of the four types of fabrics were obviously different. The COT fabric burned vigorously, swiftly, and thoroughly, leaving almost no char residues after burning. In addition, its afterflame time (2.9 s) and afterglow time (17.2 s) were the highest among the tested fabrics. However, the fireproof cotton fabric (COT-SC-Ba) did not burn visibly and generated a full char layer when exposed to flame. Moreover, its char length was measured to be merely 75 mm, and no afterflame or afterglow times were observed. This means that the COT-SC-Ba fabric could be subjected to vertical combustion tests, and the results could be mirrored in the alterations to the char layer’s appearance and a shortened char length, as well as the absence of afterflame or afterglow times. Certainly, it is inevitable to deduce that the COT-SC-Ba fabric, characterized by a distinct egg-box configuration resulting from the chelation between multiple -SO_3_^−^ and Ba^2+^ ions, acted as a barrier layer to shield the inner fibers effectively from heat, imparting exceptional flame retardancy to the COT fabric. In addition, the fire retardancy of the washed COT-SC-Ba fabric was examined. After five washing cycles, the COT-SC-Ba fabric exhibited slower burning rates and developed a more intact char layer, as shown in Figure 8c, in contrast to our previous research [21]. This may be attributed to the greater stability of the S-C bond compared to that of the C=N bond and to the good adsorption of the introduced SO_3_^−^ groups themselves. In parallel, upon re-chelating the washed COT-Glu-Ba with Ba^2^⁺ ions, its outstanding fire resistance was restored. This further confirms that COT fabric, which has undergone diverse modifications and crosslinking reactions with Ba^2^⁺ ions, possesses the ability to regain its flame retardancy.

**Figure 9:**
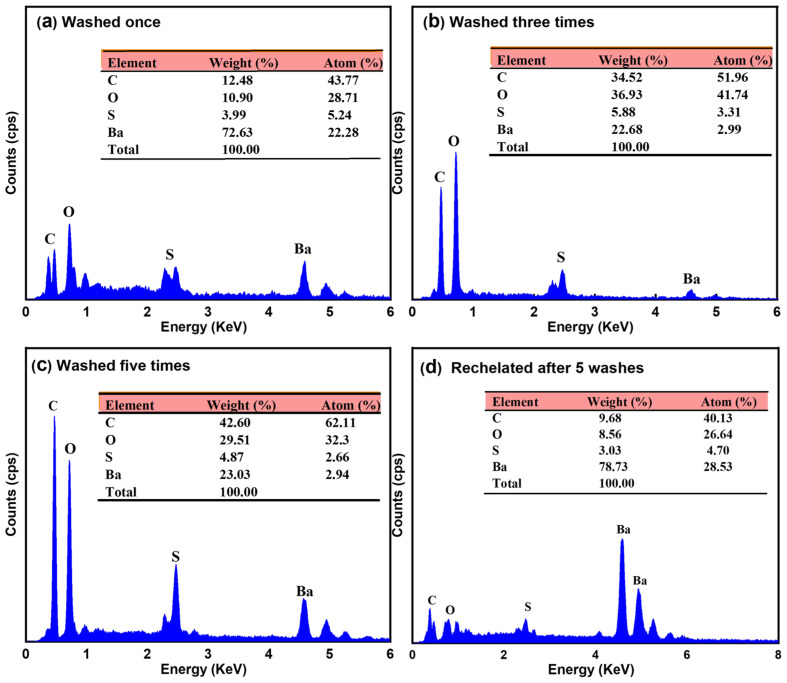
Analysis of washing resistance for flame-retardant fabrics by EDS results: (**a**) COT-SC-Ba fabric washed once, (**b**) COT-SC-Ba fabric washed three times, (**c**) COT-SC-Ba fabric washed five times, (**d**) washed and re-chelated COT-SC-Ba.

As illustrated in Table 3, the limiting oxygen index (LOI) for COT-SC-Ba reached 34.4%, marking a notable 16.8% increase compared to that of the COT. However, after five washing cycles, the LOI of COT-SC-Ba decreased to 21.6%. Fortunately, upon washing and re-chelating the fabric, the LOI of COT-SC-Ba rebounded to 29.7%, highlighting its outstanding and renewable flame-resistant properties. This recoverability aligns with our previous research [21]. According to the elemental content analysis of EDS (Figure 9), the atomic percentages of the Ba element decreased to 22.28%, 2.99%, and 2.94% after washing the flame-retardant cotton fabric one, three, and five times, respectively. Notably, the loss of Ba^2+^ during the first wash was significantly higher than during the subsequent washes. After three washes, the loss amount remained relatively constant at about 2.9%. Fortunately, the five-times-washed flame-retardant cotton fabric was re-chelated with Ba^2+^, whose Ba atomic percentage was improved from 2.94 to 28.93%. This increased amount of Ba was helpful to form the dense protective char layer during combustion. Therefore, the LOI result is consistent with the element analysis of EDS and the photograph of the vertical combustion.

Superior flame-retardant cotton fabrics may be obtained because the Ba^2+^ ions adhered to the surface of the fiber are capable of reacting with the released H_2_O, CO_2_, SO_2_, etc., at high temperatures to generate BaSO_4_, BaO, BaCO_3_, etc. These compounds covered the surface of the fabric as it burned, forming a protective barrier that effectively blocked or slowed down the direct contact and interaction between the condensed-phase material and the fire source. The SEM images also revealed the formation of a compact layer of solid residues, effectively hindering the passage of heat and oxygen, ultimately conferring fire resistance to the COT fabric. Simultaneously, the decomposition of BaSO_4_/BaO/BaCO_3_ could absorb considerable heat from the surroundings, thereby lowering the temperature of the fabric surface. Additionally, nonflammable gasses like CO_2_, SO_2_, and SO_3_ were generated due to the presence of S and C in COT-SC-Ba, which further diluted the O_2_ necessary for combustion. Ba^2+^ ions could facilitate the decomposition of sulfonic acid groups grafted onto the fiber macromolecule and enhance the development of a protective carbonized layer, which shielded the internal fibers from heat. The combined presence of Ba^2+^ ions and the S element from sulfurous acid groups played a primary fireproof role in the condensed phase, significantly enhancing the fire resistance of COT fabrics.

**Figure 10:**
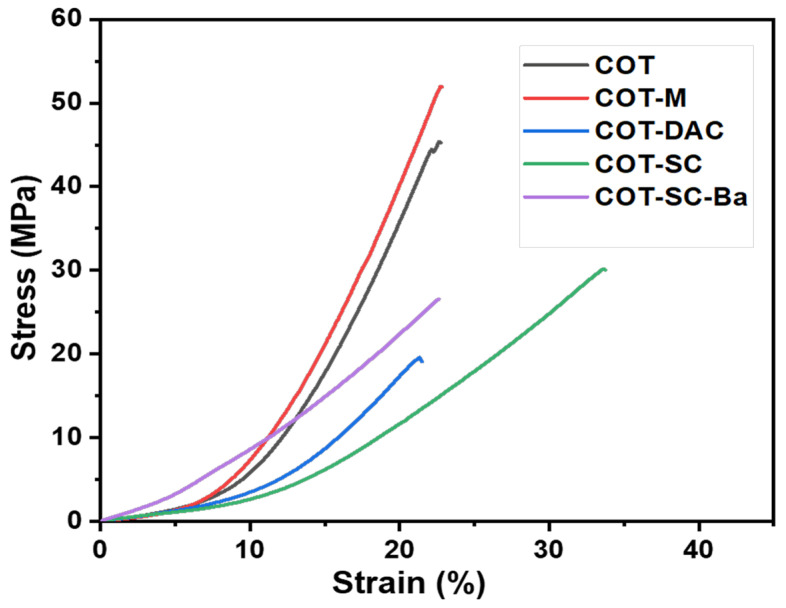
Stress–strain curves of COT and a series of modified cotton fabrics.

From the stress–strain behavior of the various samples shown in Figure 10, as well as the data provided in Table 4, it can be found that the elastic modulus of COT was 6.18 MPa, which was improved to 26.34 MPa after flame-retardant treatment, representing an increase by 326.2%. However, the tensile strength and breaking strain of COT decreased from by 49.88% 44.3 ± 0.9 MPa to 22.2 ± 4.07 MPa and increased by 8.01% from 22.98 ± 0.25% to 24.82 ± 2.19%, respectively. This result could be attributed to the chelation effect of -SO_3_^−^ and Ba^2+^, but the enhanced interface bonding strength between Ba^2+^ and the cotton macromolecule was not sufficient to compensate for the damage caused by the chemical agents. Consequently, it could provide the COT-SC-Ba fabric with a better ability to resist elastic deformation.

The original cotton (COT) fabric has a whiteness level of 79.44%, and after flame resistance treatment, this whiteness decreased by only 3.5%, demonstrating that the cotton’s whiteness was successfully preserved following the final modification. Furthermore, the testing of the WG revealed a significant increase of 22.38% after modification.

**Figure 11:**
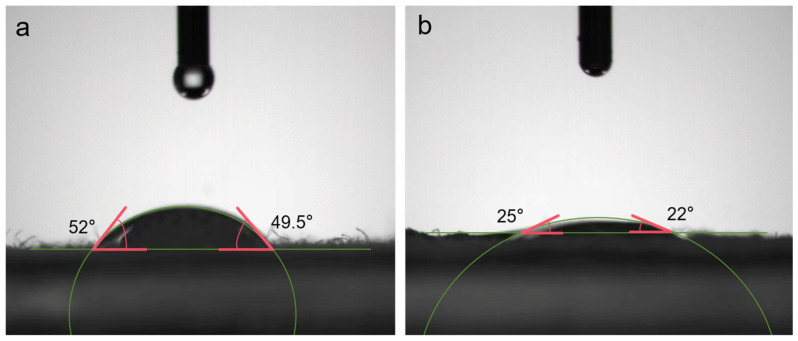
Water contact angle images: (**a**) COT and (**b**) COT-SC-Ba.

As depicted in Figure 11, the water contact angle for cotton fabric was 50.75 ± 3.8°, showing excellent hydrophilicity. After the fireproof modification, this angle decreased by a value of 27.25 ± 2.6°, suggesting that the wettability of flame-resistant cotton fabric did not become worse, conversely, improving the wettability. The likely cause may be ascribed to the superior hygroscopic properties of the sulfonic acid groups [65]. In conclusion, physical tests further suggested that the capacity for barium ions to chelate with -SO_3_^−^ endowed the COT with outstanding fire resistance, while simultaneously preserving the fabric’s other inherent properties in an excellent state (see Figure 11).

## 3. Experimental Setup

### 3.1. Materials and Reagents

The sodium hydroxide (NaOH) used in this context was sourced from Jiangsu Tongsheng Chemical Reagent Co., Ltd., located in Yixing, China. Glacial acetic acid (HAc, C_2_H_4_O_2_), glycerol (GL, C_3_H_8_O_3_) and Absolute ethanol (abs, C_2_H_6_O) were sourced from Sinopharm Chemical Reagents Co., Ltd., located in Shanghai, China. Sodium periodate (NaIO_4_) was supplied by Shanghai Shanpu Chemical Co., Ltd., located in Shanghai, China. Sodium bisulfite (SBS, NaHSO_3_) and barium chloride (BaCl_2_·2H_2_O) were bought from Macklin Biochemical Co., Ltd., located in Shanghai, China. All of the above chemical reagents were of analytical grade purity and were utilized directly without any additional purification steps. The cotton fabric, provided by the Shanghai Textile Industry Technical Supervision Institute, located in Shanghai, China, was woven using a plain weave pattern. The fabric is characterized by a linear density of 20 tex for both its warp and weft yarns, features a fabric count of 300 yarns/10 cm in both directions, and weighs 112 g/m^2^.

### 3.2. Preparation of Flame-Retardant Fabrics

**Scheme 1:**
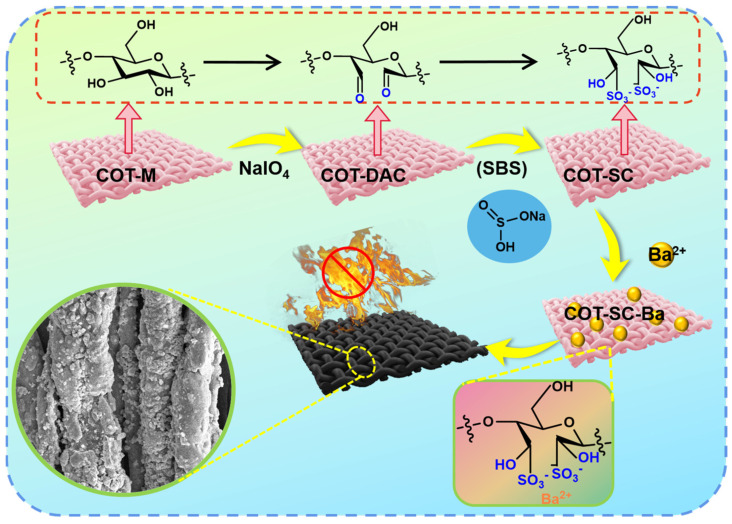
Diagrammatic illustration of the procedure for altering cotton fabrics.

The steps in introducing sulfonate groups and then chelating Ba^2+^ ions onto cotton fabrics are outlined in Scheme 1. Initially, a cotton fabric piece (320 mm × 90 mm) was dipped in a 25 wt.% NaOH solution with a bath ratio of 1:25, while being continuously stirred for 10 min at ambient temperature. Excess NaOH was then neutralized using a 0.5 wt.% (5 g/L) acetic acid solution for 30 min. Afterward, the fabric was washed while stirring for 10 min and dried at 65 °C to produce alkali-treated cotton fabric (COT-M). Next, this alkali-treated fabric underwent oxidation by being immersed in a 0.2 mol/L NaIO_4_ solution under vigorous stirring in the dark at 40 °C for 1 h. Following this, the fabric was soaked in a 0.1 mol/L solution of glycerol with a bath ratio of 1:17 at ambient temperature for 30 min with stirring, and then dried to yield the oxidation product, named COT-DAC. Subsequently, a solution of 140mL, composed of a 50:50 mixture of ethanol and water, was prepared and then combined with 10g of sodium bisulfite (NaHSO₃) and the COT-DAC fabric. This mixture reacted for 10 h, subsequently undergoing washing and drying processes to obtain the fabric grafted with -SO_3_^−^ groups, designated as COT-SC. Finally, the COT-SC fabric was immersed in a saturated barium chloride solution for 0.5 h to allow for chelation. Afterward, it was thoroughly washed with deionized water to eliminate any unchelated barium chloride (BaCl_2_), and then dried to yield the fireproof cotton fabric (COT-SC-Ba). The formula utilized for calculating the weight gain (WG) of the COT-SC-Ba fabric is provided below:

WG (%) = (m_FR_ − m_0_)/m_0_ × 100% (1)

where m_0_ and m_1_ denote the weight of the cotton fabric prior to and following the flame-retardant treatment, respectively.

### 3.3. Characterization

The powder of samples such as untreated cotton, various treated cottons, and carbon residues were each co-grinded with potassium bromide, followed by pressing them into tablets and analyzing them using Fourier Transform Infrared (FTIR) spectroscopy (NEXUF-670, Thermo Nicolet Corporation, Waltham, MA, USA). The frequency step in the measurements was set at 2 cm^−1^, with a scanning range of 4000–400 cm^−1^. The X-ray Photoelectron Spectrometer (ESCALAB 250Xi, ThermoFisher Scientific, Carlsbad, CA, USA) was utilized to investigate the composition of the samples. The operating voltage was set at 12 kV, with a working current of 15 mA. The excitation radiation employed was Al Kα at 1486.6 eV, and the binding energy was calibrated to the C 1s line at 284.6 eV. The crystalline structure of the samples prepared in the experiment and burned through the VBT was analyzed using the XRD (X’Pert3 Powder, PANalytical B.V., Almelo, The Netherlands), corresponding to the following parameters: Cu Kα radiation with λ = 1.5418 Å, a 40 kV tube voltage, and a 0.026° scan step. The SEM (Nova NanoSEM 450, FEI Company, Hillsboro, OR, USA) was utilized to analyze the micromorphology of COT, COT-SC-Ba, and carbon residue after the VBT with an accelerating voltage of 5 kV and a magnification range of 500–5,000×. Simultaneously, EDS (Aztec X-MaxN80, Oxford Instruments, Abingdon, UK) testing was conducted to examine the elemental composition and content for a variety of samples.

The fire performance of the samples in an actual fire environment was evaluated using an FTT-i-Cone 0402 cone calorimeter (Fire Testing Technology Limited, East Grinstead, UK) according to ISO5660 [66]. The cone calorimeter was used to examine the dimensions of a 100 mm × 100 mm × 3 mm sample, and the treated cotton textiles were placed under a radiant cone with an external heat flux of 35 kW/m^2^. The thermal pyrolysis behavior of the samples before and after treatment was tested through TG analysis (STA6000, Perkin Elmer, Waltham, MA, USA) at a heating rate of 10 °C/min with the temperature ranging from 40 and 800 °C in both N_2_ and air. LOI and vertical combustion tests were used to assess the flame resistance and durability of the samples. The HC-2 oxygen index tester, manufactured by Ruixinjie Instrument in China, was used to determine the LOI value of the sample, which measured 150 mm × 50 mm. Additionally, the vertical burning experiment was carried out on a specimen sized 89 mm × 300 mm using the YG(B)815D-I tester, produced by Darong Textile Standard Instrument in Wenzhou, China.

The tensile strength of the various samples was measured under normal conditions using the CMT4304 strength tester, manufactured by MTS Systems Co., Ltd., located in Shenzhen, China. The whiteness of the relevant samples was evaluated with the WSD-3 automatic whiteness meter, produced by Beijing Kangguang Optical Instrument Co., Ltd. in Beijing, China. To assess the wettability of COT and COT-SC-Ba, static contact angle measurements were conducted using the JC2000D3 tester, supplied by Zhongchen Digital Technical Equipment Co., Ltd., based in Shanghai, China. The durability testing of the final modified fabric was performed in accordance with the AATCC 61-2013 standard [67] using the SW-12AII washing fastness tester, manufactured by Darong Textile Standard Instrument in Wenzhou, China. During the test, the fabric underwent repeated washing cycles in a rotating, sealed stainless steel container with a 500 mL capacity. The washing solution consisted of 0.15 wt.% soap powder sourced from the Shanghai Textile Industry Technical Supervision Institute. Each washing cycle was conducted at a temperature of 49 °C, with a bath ratio of 1:50, and lasted for 15 min.

## 4. Conclusions

A novel, simple, and eco-friendly process for the production of a fireproof cotton fabric has been successfully achieved. This process constructed a distinctive skeletal network on both the exterior surface and the interior of the cotton fiber, strengthening the bonds between the macromolecules. The thermal stability and flame retardancy of the COT-SC-Ba fabric significantly increased, as evidenced by tests such as the CCT, TG, LOI, and VBT. The CCT showed that the TTI value increased by 8 s, and the PHRR, TSR, EHC, and CO/CO_2_ ratio of COT-SC-Ba decreased by 39.2%, 96.4%, 20.1% and 27.8%, respectively. TG tests exhibited that the peak weight loss rate (Rmax) decreased by 35.5% in air, while the Rmax remained nearly unchanged in N_2_. Furthermore, the mass of char residues greatly increased whether in air or N_2_. The LOI value reached 34.4%. In addition, the char length in the VBT was merely 75 mm with no afterflame or afterglow time after a burning of 12 s, which showed the outstanding self-extinguishing property. The effective flame retardancy observed was due to the synergistic action of the introduced -SO_3_^−^ ions and the chelated Ba^2+^ ions. During burning, the chelated Ba^2+^ ions reacted with released gasses to form a dense charcoal layer enveloping the cotton fabric, which provided a protective layer to hinder heat, fire, and combustible gas so as to protect the internal fibers from thermal decomposition. This treatment strategy not only displayed remarkable fire resistance but also maintained its pristine whiteness, desired tensile strength, and superior moisture absorption capability. This research developed a highly effective approach to manufacturing flame-retardant cotton fabrics that are free from halogen, phosphorus, and formaldehyde, while preserving their intrinsic properties.

## Figures and Tables

**Table 1:** Cone calorimeter test data of COT and COT-SC-Ba.

Samples	TTI (s)	PHRR (kW/m^2^)	TPHRR (s)	THR (MJ/m^2^)	CO_2_/CO (%)	FIGRA (kW/m^2^·s)	TSR (m^2^/m^2^)	Av-EHC (MJ/Kg)
COT	15	250.50	32	7.34	15.36	34.17	37.02	19.36
COT-SC-Ba	23	152.37	35	8.48	11.09	17.97	1.32	15.47

**Table 2:** Thermogravimetric analysis data of COT and COT-SC-Ba in air and N_2_.

Atmosphere	Sample	T_onset_ (°C)	T_max_ (°C)	R_max_ (%/min)	Residual Mass at 800 °C (wt.%)
Air	COT	310.6	339.2	18.3	0.02
	COT-SC-Ba	261.5	315.8	11.8	26.9
N_2_	COT	322.6	357.9	14.6	8.05
	COT-SC-Ga	266.2	315.1	14.7	26.8

**Table 3:** Data on the LOI and vertical burning tests for four types of fabric in air.

Sample	LOI (%)	Afterflame Time (s)	Afterglow Time (s)	Char Length (cm)
COT	17.6 ± 0.6	2.9 ± 0.3	17.2 ± 1.2	≥34
COT-SC-Ba	34.4 ± 0.4	0	0	7.5 ± 0.5
COT-SC-Ba washed in 5 cycles	21.6 ± 0.5	3.2 ± 0.2	8.9 ± 2.2	≥34
Washed and re-chelated COT-SC-Ba	29.7 ± 0.7	3.1 ± 0.3	131.4 ± 9.7	7.8 ± 0.6

**Table 4:** Mechanical properties and whiteness of COT and various modified cotton fabrics.

Sample	Elasticity Modulus (MPa)	Breaking Strain (%)	Tensile Strength (MPa)	Whiteness Index (%)
COT	350.57 ± 13.78	22.98 ± 0.25	44.3 ± 0.9	79.44
COT-M	290.76 ± 28.84	37.44 ± 3.47	48.47 ± 3.28	/
COT-DAC	152.13 ± 3.94	20.2 ± 2.78	17.48 ± 1.87	/
COT-SC	124.88 ± 3.27	35.29 ± 1.71	30.22 ± 0.23	/
COT-SC-Ba	85.8 ± 16.93	24.82 ± 2.19	22.2 ± 4.07	76.75

## Data Availability

All information generated or meticulously analyzed throughout the course of this study has been comprehensively included in this published article.
